# The effects of diet induced obesity on breast cancer associated pathways in mice deficient in SFRP1

**DOI:** 10.1186/1476-4598-13-117

**Published:** 2014-05-22

**Authors:** Kelly J Gauger, Lotfi M Bassa, Elizabeth M Henchey, Josephine Wyman, Jennifer Ser-Dolansky, Akihiko Shimono, Sallie S Schneider

**Affiliations:** 1 Pioneer Valley Life Sciences Institute, Baystate Medical Center, 3601 Main St, Springfield, MA 01199, USA; 2 Biology Department, University of Massachusetts, Amherst, MA 01003, USA; 3 Veterinary and Animal Sciences, University of Massachusetts, Amherst, MA 01003, USA; 4 TransGenicInc., Kobe 650-0047, Japan

**Keywords:** Sfrp1, Obesity, Breast cancer, Wnt signaling, Apoptosis, p53, RANKL

## Abstract

**Background:**

Secreted frizzled-related proteins (SFRPs) are a family of proteins that block the Wnt signaling pathway and loss of Sfrp1 expression is observed in breast cancer. The molecular mechanisms by which obesity contributes to breast tumorigenesis are not well defined, but involve increased inflammation. Mice deficient in Sfrp1 show enhanced mammary gland inflammation in response to diet induced obesity (DIO). Furthermore, mammary glands from *Sfrp1*^*−/−*^ mice exhibit increased Wnt signaling, decreased cell death responses, and excessive hyper branching. The work described here was initiated to investigate whether obesity exacerbates the aforementioned pathways, as they each play a key roles in the development of breast cancer.

**Findings:**

Wnt signaling is significantly affected by DIO and *Sfrp1*^*−/−*^ loss as revealed by analysis of *Myc* mRNA expression and active β-catenin protein expression. Furthermore, *Sfrp1*^*−/−*^ mice fed a high fat diet (HFD) exhibit an increase in mammary cell proliferation. The death response is also impaired in the mammary gland of *Sfrp1*^*−/−*^ mice fed a normal diet (ND) as well as a HFD. In response to γ-irradiation, mammary glands from *Sfrp1*^−/−^ mice express significantly less *Bax* and *Bbc3* mRNA, caspase-3 positive cells, and p53 protein. The expression of *Wnt4* and *Tnfs11* are critical for normal progesterone mediated mammary gland development and in response to obesity, *Sfrp1*^−/−^ mice express significantly more *Wnt4* and *Tnfs11* mRNA expression. Evaluation of progesterone receptor (PR) expression showed that DIO increases the number of PR positive cells.

**Conclusions:**

Our data indicate that the expression of *Sfrp1* is a critical factor required for maintaining appropriate cellular homeostasis in response to the onset of obesity.

## Findings

Obesity has increased with an alarming rate in the United States. It is estimated that by 2015, 75% of the population will be either overweight or obese. This fact has elicited a serious public health concern since obesity increases the incidence, progression, and mortality from breast cancer [1]. Cancer results from cellular mutations that enhance proliferation and decrease programmed cell death [2]. Our earlier published studies focused on the role a tumor suppressor gene, secreted frizzled related protein 1 (Sfrp1), plays in mammary gland development and cell death. We revealed that loss of *Sfrp1* alters the growth and behavior of mammary epithelial in such a manner that they exhibit characteristics of breast cancer cells [3,4]. Moreover, *Sfrp1* plays a critical role in mediating the mammary epithelial cellular apoptotic response to DNA damage *in vivo*[5]. Recently, we found that mice deficient in *Sfrp1* (*Sfrp1*^−/−^) fed a high fat diet (HFD) exhibit a significant increase in body mass, body fat percentage, as well as adipocyte size and have elevated fasting glucose levels and impaired glucose clearance abilities [6]. Additionally, the inflammatory state of mammary glands from *Sfrp1*^−/−^ mice fed a HFD is elevated as revealed by increased macrophage infiltration and pro-inflammatory cytokine expression [6] Considering the connection between obesity and inflammation, loss of *Sfrp1* may be a critical early event in obesity associated breast cancer initiation.

**Figure 1:**
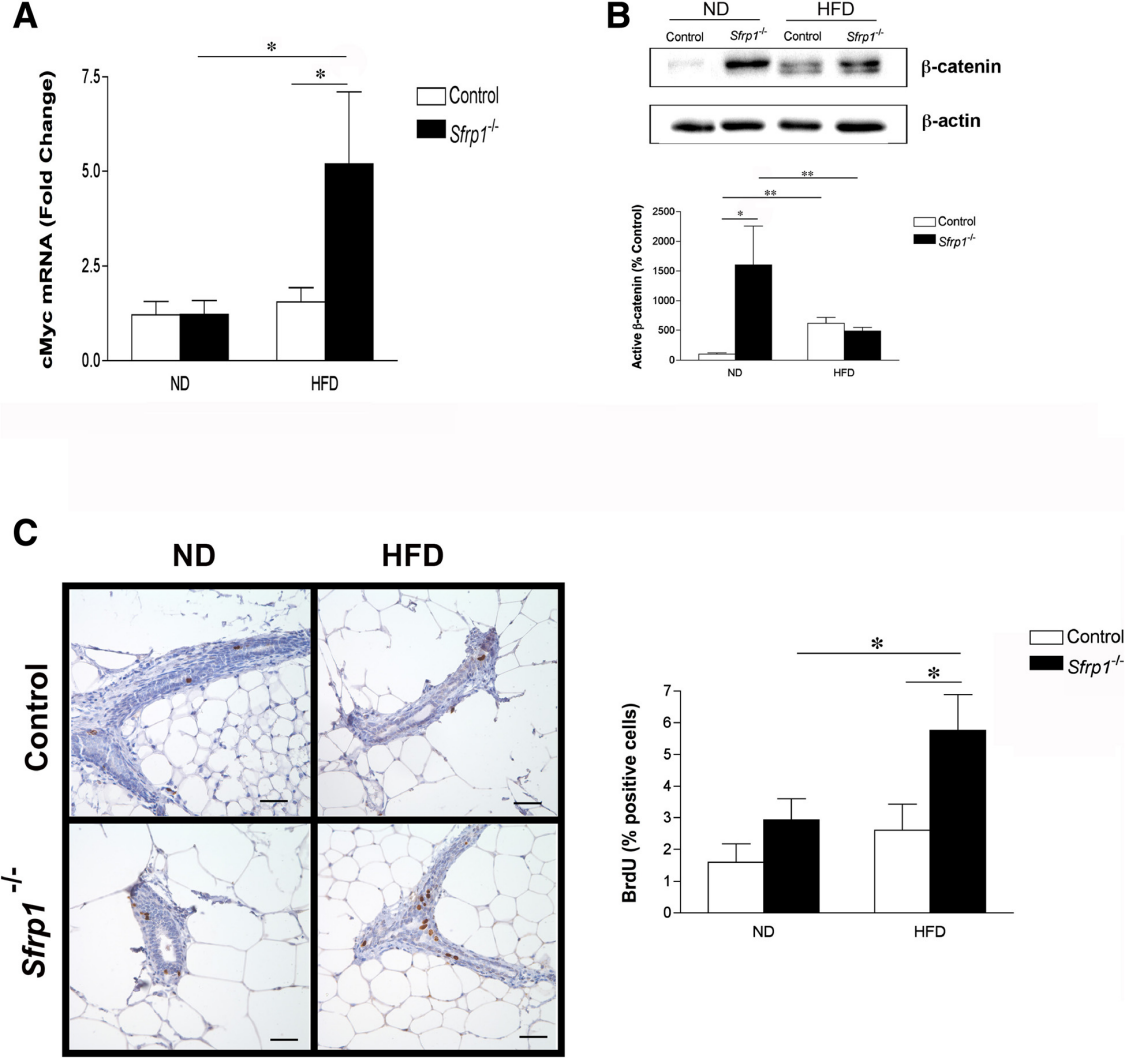
**Loss of *Sfrp1 *increases Wnt signaling and cellular proliferation in response to DIO the murine mammary gland. (A)** Total RNA was harvested from 5^th^ inguinal mammary glands and employed for real-time PCR analysis of *Myc* gene expression (n = 6/genotype). The results shown represent experiments performed in duplicate and are normalized to the amplification of *β-Actin* mRNA. Bars represent mean ± SEM of the difference in fold change compared with control ND fed mice. **(B)***Upper panel,* Mammary gland lysates were analyzed for non-phospho (active) β-catenin and β-actin protein expression by western blot. *Lower panel,* Band density was quantified and bars represent mean ± SEM of % control ND fed mice. **(C)***Left panel*, 3^rd^ & 4^th^ inguinal mammary gland sections were subjected to immunohistochemical analysis, stained for BrdU (brown chromogen), and representative images were captured at 400X are displayed for mice in each treatment group (scale bar 50 μm). *Right panel*, BrDU-stained cells were counted out for each mammary gland (n = 6/genotype) and bars represent mean ± SEM % BrdU-positive cells. (*p < 0.05, significantly different from control mice fed a ND using Bonferroni’s *t* test after a two-way ANOVA).

The Wnt family of secreted proteins is implicated in the regulation of cell fate during development, as well as in cell proliferation, morphology, and migration [7]. The best characterized Wnt pathway is the canonical Wnt/β-catenin pathway whereby Wnt signaling leads to the stabilization of β-catenin and activation of β-catenin-responsive gene expression. Sfrp1 antagonizes Wnt signaling by binding to Wnt ligands and preventing ligand-receptor interactions and signal transduction [8]. Indeed, loss of SFRP1 increases Wnt signaling in mammary epithelial cells [3], a deleterious effect considering that inappropriate activation of the Wnt/β-catenin pathway contributes to the development of breast cancer [7]. To determine whether increased adiposity exacerbates the effect of *Sfrp1* loss on Wnt/β-catenin signaling, we measured the mRNA expression of the β-catenin target gene, *Myc*, in control and *Sfrp1*^*−/−*^ mice [9] (Additional file 1: Figure S1) fed a normal diet (ND) and HFD. A two-way ANOVA revealed that *Myc* was significantly affected in response to *Sfrp1* loss on the HFD (F_1,17_ = 5.17; P < 0.05; F_1,17_ = 5.23; P < 0.05). In addition, there was a significant interaction between these two main effects (F_1,17_ = 7.34; P < 0.05) (Figure 1A). These findings are consistent with our recently published results demonstrating that *Axin2*, a hallmark Wnt target gene, is significantly elevated in the mammary gland of *Sfrp1*^*−/−*^ mice fed a HFD [6]. To investigate whether Wnt signaling is activated in the absence of *Sfrp1*, we employed western blot analysis with a non-phospho (active) β-catenin antibody (Figure 1B, *upper panel*). Densitometry measurements revealed that the active form of β-catenin was significantly upregulated in response to *Sfrp1* loss (F_1,10_ = 8.50; P < 0.05) as well as the HFD (F_1,10_ = 5.94; P < 0.05), but there was no interaction between these two main effects (F_1,10_ = 1.15; P > 0.05) (Figure 1B). We show that in response to DIO, β-catenin activity was significantly increased, but the absence of Sfrp1 did not further enhance the expression of active β-catenin. These data may be partially explained by published findings and our previous results which demonstrate that adiposity increases the expression of other Wnt signaling antagonists, including Sfrp5, and thus may act to diminish the effect of Sfrp1 loss on β-catenin activity [10,11]. Given the role Wnt/β-catenin plays in cellular proliferation, mice were injected with BrdU to evaluate the effect of *Sfrp1* loss and diet induced obesity (DIO) on proliferation. We reveal that the percentage of BrdU positive epithelial cells was significantly increased in response to *Sfrp1* loss (F_1,18_ = 7.02; P < 0.05) as well as the HFD (F_1,18_ = 5.10; P < 0.05), but there was no interaction between these two main effects (F_1,18_ = 1.13; P > 0.05) (Figure 1C). Although both DIO and *Sfrp1* loss exhibited effects on their own that could participate in an increased risk for cancer, the expression of *Myc* was enhanced by the two main effects together suggesting that a HFD and *Sfrp1* loss, through methylation or mutation, could drive the expression of *Myc* to very high levels and thus work together to promote cancer risk. Thus, in the context of obesity, *Sfrp1* expression is especially important in preventing aberrant Wnt signaling.

**Figure 2:**
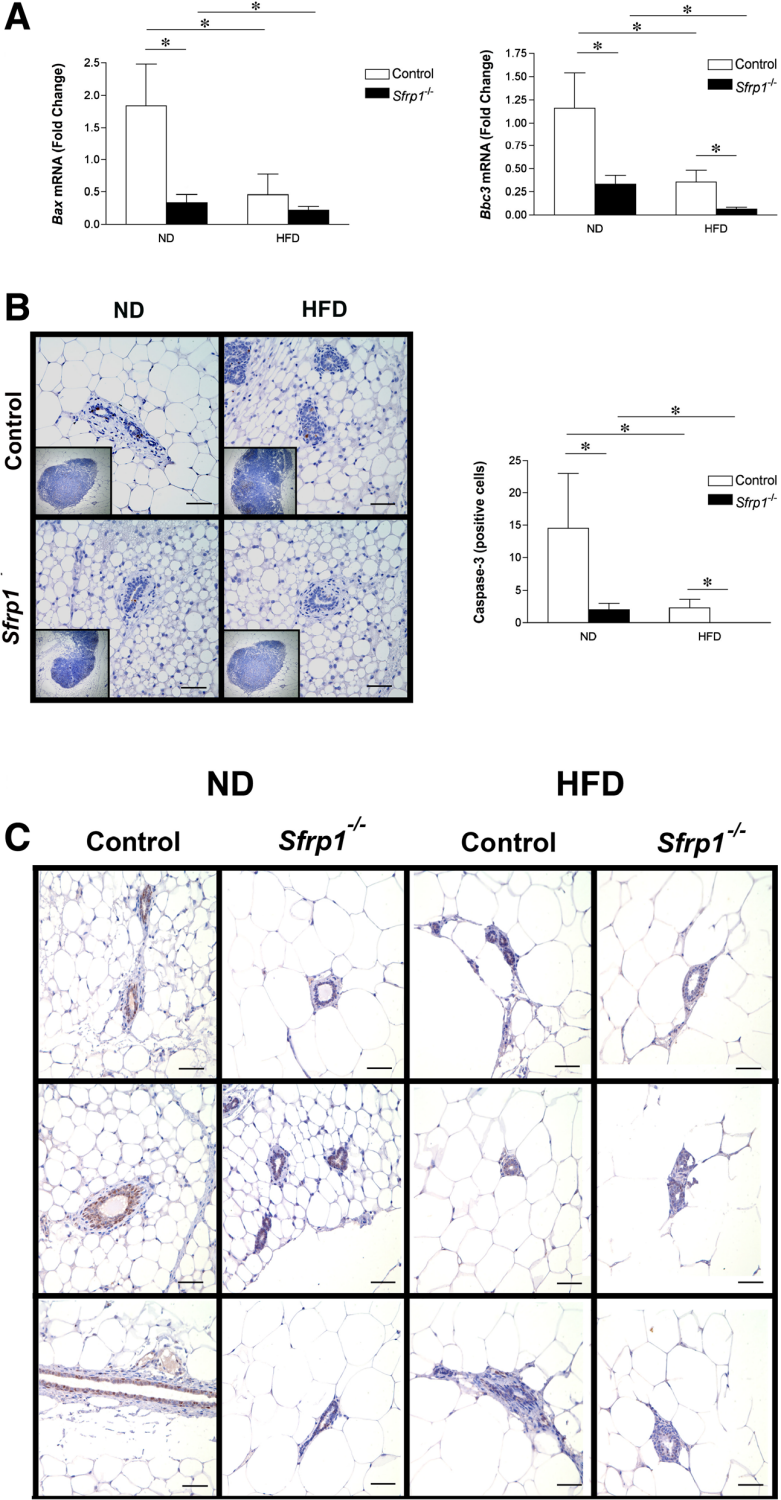
**Mammary glands from *Sfrp1***^***−/− ***^**exhibit a decrease and cell death signals in response to γ-irradiation. (A)** For real-time PCR analysis of *Bax* and *Bbc3* gene expression, total RNA was isolated from the mammary glands of control and *Sfrp1*^−/−^ female mice fed a ND and HFD 6 hours following 5 Gy whole body irradiation (n = 3/geneotype). The results shown represent experiments performed in duplicate and are normalized to the amplification of *β-Actin* mRNA. Bars represent mean ± SEM of the difference in fold change compared with control ND fed mice. **(B)***Left panel,* 3^rd^ & 4^th^ inguinal mammary gland sections were subjected to immunohistochemical analysis, stained for cleaved caspase-3 (brown chromogen), and images were captured at 400X. *Inset*, In addition to capturing 400X photographs of mammary gland ducts, lymph nodes were imaged as a positive control (40X). *Right panel*, The total number of cleaved caspase-3 positive cells was counted for each mammary gland (n = 3/genotype) and bars represent mean ± SEM cell number. **(C)** 3^rd^ & 4^th^ inguinal mammary gland sections were subjected to immunohistochemical analysis, stained for p53 (brown chromogen), and representative images were captured at 400X (scale bar 50 μm). Pictures illustrate the staining results obtained from each γ-irradiated mouse in the study. (*p < 0.05, significantly different from control mice fed a ND using Bonferroni’s *t* test after a two-way ANOVA).

*Sfrp1* downregulation leads to a resistance to anoikis (apoptosis triggered by loss of attachment) [3]. Resistance to death triggers, due to mutations or loss of attachment, is an important capability for metastasis to occur by allowing cellular survival until colonization in a distant location. *Sfrp1* has been shown to induce apoptosis in numerous tissues [3,12-15] and loss of *Sfrp1* significantly impacts apoptotic related gene expression as well as activity [5] suggesting a causative role for reduced *Sfrp1* in premalignant breast changes leading to tumor progression. Given that loss of *Sfrp1*^*−/−*^ mice are more resistant to γ-irradiation induced cell death [5], we exposed control and *Sfrp1*^*−/−*^ mice fed a ND and a HFD to 5Gy whole body irradiation to assess whether loss of *Sfrp1* in our DIO model inhibits death responses. We first measured the expression of *Bax*, a major mediator of pro-apoptotic activity in mammary epithelial cells. Real-time PCR analysis demonstrated that that the expression of *Bax* mRNA was significantly affected by *Sfrp1* loss (F_1,7_ = 9.03; P < 0.05) and the HFD (F_1,7_ = 6.76; P < 0.05) and there was also an interaction between these two main effects (F_1,7_ = 4.83; P < 0.05) (Figure 2A). Additionally, we assessed the expression of *Bbc3* (*aka* PUMA), a key p53 transcriptional target [16]. Our data show that *Bbc3* is significantly repressed in response to *Sfrp1* loss (F_1,7_ = 6.1; P < 0.05) as well as the HFD (F_1,7_ = 5.57; P < 0.05), but there was no interaction between these two main effects (F_1,7_ = 1.41; P > 0.05) (Figure 2A). Caspase-3 is a key intracellular effector of apoptosis by cleaving critical protein substrates required for apoptotic cell death [17]. Immunohistochemical analysis of the cleaved (activated) form of caspase-3 revealed that the immune cells within the lymph node of both genotypes underwent apoptosis serving as an excellent internal positive control for our assay (Figure 2B, *left panel*). The total number of cleaved-caspase-3 positive luminal epithelial cells were quantified and our data reveal that there was a significant reduction in caspase-3 positive cells of in response to *Sfrp1* loss (F_1,7_ = 6.37; P < 0.05) as well as the HFD (F_1,7_ = 5.81; P < 0.05), but there was no interaction between these two main effects (F_1,7_ = 2.99; P > 0.05) (Figure 2B, *right panel*). Finally, we wished to look at the effect DIO in *Sfrp1*^*−/−*^ mice on p53 expression. Consistent with our earlier findings, there are less intensely stained nuclei in *Sfrp1*^*−/−*^ mice compared to control mice fed a ND. Additionally, p53 expression is diminished in animals fed a HFD independent of geneotype (Figure 2C). Although work confirms previous studies which demonstrate that obesity inhibits cell death responses [18,19], these novel findings are the first to demonstrate that the DIO diminishes mammary epithelial cell death and that the expression of p53 is repressed by DIO in the mammary gland. These data may be partially explained by the elevated insulin observed levels in these animals [6] as insulin has been shown to reduce apoptosis in mammary epithelial cells *in vitro*[20]. Taken together, our results suggest a possible mechanism by which obesity promotes mammary tumorigenesis.

**Figure 3:**
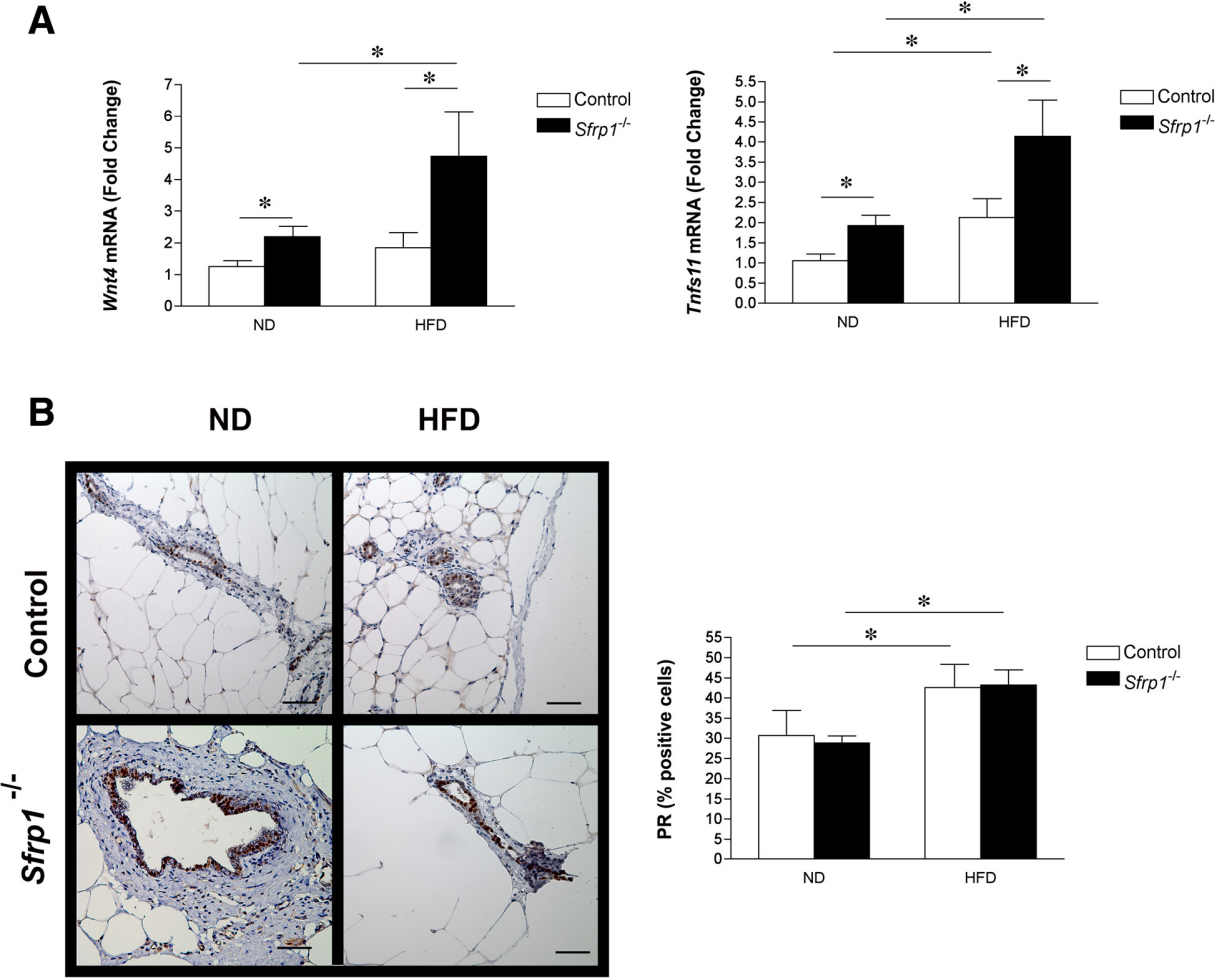
**Genes involved in mammary gland development are aberrantly up-regulated in *Sfrp1***^***−/− ***^**mice in response to DIO. (A)** Real-time PCR analysis of *Wnt4* and *Tnfs11*gene expression was carried out (n = 6/genotype). The results shown represent experiments performed in duplicate and are normalized to the amplification of *β-Actin* mRNA. Bars represent mean ± SEM of the difference in fold change compared with control ND fed mice. **(B)***Left panel*, 3^rd^ & 4^th^ inguinal mammary gland sections were subjected to immunohistochemical analysis, stained for PR (brown chromogen), and representative images were captured at 400X are displayed for mice in each treatment group (scale bar 50 μm). *Right panel*, PR-stained cells were counted in each mammary gland (n = 6/genotype) and bars represent mean ± SEM % PR-positive cells. (*p < 0.05, significantly different from control mice fed a ND using Bonferroni’s *t* test after a two-way ANOVA).

We previously showed that *Sfrp1*^*−/−*^ mice exhibit a higher density of ducts with distinct alveoli present throughout the mammary gland with focal ductal epithelial hyperplasia [4]. These data are fully consistent with previous studies showing that upregulation of the Wnt/β-catenin pathway and activation of β-catenin in mice induces precocious lobulo-alveolar hyperplasia [21,22]. Constitutive expression of Wnt4 in the virgin mammary gland also induces structures with a morphology similar to that seen in pregnancy [23] and *Wnt4* is significantly up-regulated in pubescent *Sfrp1*^*−/−*^ mice. We employed real-time PCR analysis to examine the effects of *Wnt4* in *Sfrp1*^*−/−*^ mice in response to DIO and a two-way ANOVA revealed that *Wnt4* is significantly increased in response *Sfrp1* loss (F_1,19_ = 6.44; P < 0.05) as well as the HFD (F1_1,19_ = 4.34; P < 0.05), but there was no interaction between these two main effects (F_1,19_ = 1.65; P > 0.05) (Figure 3A). The receptor of activated NF-κB ligand (Tnfs11 *aka* RANKL) is a critical downstream target of Wnt4 [24,25]. Transgenic overexpression of Tnfs11 into the murine mammary gland elicits ductal side branching, alveologenesis, and mammary hyperplasia [26,27]. Furthermore, SFRP1 has been shown to bind to and inhibit Tnsf11 mediated action [28], and loss of *Sfrp1* increases the expression of *Tnfs11* during puberty. Here we show that *Tnfs11* was significantly increased in response to *Sfrp1* loss (F_1,18_ = 10.7; P < 0.05) as well as the HFD (F1_1,18_ = 13.7; P < 0.05), but there was no interaction between these two main effects (F_1,19_ = 1.65; P > 0.05) (Figure 3A). Since *Wnt4* and *Tnfs11* are downstream effectors of progesterone signaling [29], we evaluated progesterone receptor (PR) expression in mammary ducts. Consistent with the literature, immunohistochemical analysis of PR expression illustrated that DIO increases the percentage of PR expressing cells (Figure 3B, *left panel*). The total number of PR positive luminal epithelial cells were quantified and a two-way ANOVA confirmed that there was no difference in the percentage PR expressing cells response to *Sfrp1* loss (F_1,19_ = 0.913; P > 0.05), but the HFD significantly increased PR expression (F_1,19_ = 5.55; P < 0.05), although there was no interaction between these two main effects (F_1,7_ = 0.8253; P > 0.05) (Figure 3B, *right panel*). Thus, the DIO-induced increase in PR expression may exacerbate the expression of *Wnt4* and *Tnsf11* in *Sfrp1*^*−/−*^ mice. The expression of *Sfrp1* is critical for maintaining proper mammary gland development and considering that the deleterious effects of Sfrp1 depletion are exacerbated in response to DIO, loss of *Sfrp1* in the context of obesity may be a critical event in cancer initiation. Additionally, the increased adiposity and decreased death response observed in *Sfrp1*^*−/−*^ mice may lead to increased breast cancer susceptibility. Future studies are aimed at elucidating the molecular mechanisms by which obesity and Sfrp1 downregulation affect tumorigenesis.

## Materials and methods

### Animals

This study was carried out in strict accordance with the recommendations in the Guide for the Care and Use of Laboratory Animals of the National Institutes of Health. The protocol was approved by the Baystate Medical Center Institutional Animal Care and Use Committee (Permit Number: 283237). Female129/C57Blk6 control mice (n = 20) and 129/C57Blk6 *Sfrp1*-/- mice (n = 20) were individually housed in plastic cages with food and water provided continuously, and maintained on a 12:12 light cycle. Mice (n = 10/genotype) were placed on either a normal diet [(ND) Harlan Teklad global 18% protein rodent diet (#2018) containing 2.8% fat, 18.6% protein] or placed on a high fat diet [(HFD) Bio-Serv (#F1850) containing 36.0% fat, 36.2% carbohydrate, and 20.5% protein] starting at 10 weeks of age for 12 weeks. Mice were injected 70 μg/g body weight of 5-bromo-2-deoxyuridine (BrdU; Sigma, St Louis, MO) and the glands will be harvested 24 hours later. A select number of mice from each treatment group (n = 3) were subjected to 5 Gy of whole body γ-irradiation to induce DNA damage and mammary glands were harvested 6 hours later. Animals were euthanized by CO2 followed by cervical dislocation and bled by cardiac puncture. The 3^rd^ and 4^th^ mammary glands were fixed in buffered formalin and 5th inguinal glands were flash frozen.

### Genotyping

Tail DNA was obtained from control (*Sfrp1*^*+/+*^), heterozygous ( *Sfrp1*^*-/+*^), and homozygouse knockout *(Sfrp1*^*-/-*^) mice as well as breeding pairs used to generate mice for our study as described previously [9]. PCR amplification was carried out using the Typeit Mutation Detect PCR Kit according to the manufacturer’s instructions (QIAGEN, Valencia, CA). Primmer sequences used in the reaction were as follows: *SacII* forward, 5′-GATTGGTTAACTGCGCGGCTG-3′; *SacII* reverse, 5′-GACTGGAAGCTCACGTAGTCG -3′; LacZ forward, 5′-TTCACTGGCCGTCGTTTTACAACGTC-3′; LacZ reverse, 5′-TTCACTGGCCGTCGTTTTACAACGTC-3′. SacII primers predicted to amplify 510 bp wild-type allele and LacZ primers predicted to amplify 364 LacZ target used in the generation of *Sfrp1* knockout mice. The conditions for the target DNA amplification were performed as follows: 1 cycle of 95°C for 15 min; 40 cycles each of 95°C for 30 s, 54.7°C for 1 min, and 72°C for 30 s; and 72°C for 10 min.

### RNA isolation and real-time PCR analysis

Total RNA was extracted from the 5th inguinal mammary glands using an acid-phenol extraction procedure according to the manufacturer’s instructions (Trizol, Invitrogen, Carlsbad, CA). Relative expression levels of mRNA was determined by quantitative real-time PCR using the Mx3005P™ real-time PCR system (Stratagene, La Jolla, CA) and all values were normalized to the amplification of β*-Actin.* PCR primers used for for *Sfrp1* were as follows: *Sfrp1* forward, 5′-CACAACGTGGGCTACAAGAA -3′; *Sfrp1* reverse, 5′- TCACCTCTGCCATGGTCT-3′. All other PCR primer sequences have been described previously [3-5]. The assays were performed using the 1-Step Brilliant® SYBRIII® Green QRT-PCR Master Mix Kit (Stratagene) containing 200 nM forward primer, 200 nM reverse primer, and 100 ng total RNA. The conditions for cDNA synthesis and target mRNA amplification were performed as follows: 1 cycle of 50°C for 30 min; 1 cycle of 95°C for 10 min; and 35 cycles each of 95°C for 30 s, 55°C for 1 min, and 72°C for 30 s.

### Western blot analysis

The 5th inguinal mammary glands were homogenized in cold lysis buffer [50 mM Tris-HCl, 150 mM NaCl, 100 mM NaF, 10 mM MgCl2, 0.5% NP40, protease inhibitor cocktail, and phosphatase inhibitor I and II (Sigma)]. The lysates were passed 4 times through a 26 gauge syringe, kept on ice for 30 minutes, and then centrifuged for 20 minutes at 12,000 rpms at 4°C. The supernatant was transferred to a new tube and the protein was quantified utilizing the BCA™ Protein Assay Kit (Pierce, Rockford, IL). A total of 35 μg of protein was run on a 10% SDS-Page gel and transferred to a PVDF membrane. The membrane was blocked for 45 minutes with 5% milk in tris-buffered saline containing 0.05% Tween-20 (TBS-T). The primary antibodies used in this study were as follows: Rabbit non-phospho (Active) β-catenin (Ser33/37/Thr41) (D13A1) 1:1000 (Cell Signaling Technology, Danvers, MA); Rabbit β-actin 1:2000 (Abcam, Cambridge, MA. The primary antibodies were incubated over overnight at 4°C and the secondary antibody [goat anti-rabbit IgG-HRP 1:5000 (Santa Cruz Biotechnology, Dallas, TX] was incubated for 45 minutes at room temperature. The blot was washed and developed using a Western Blot Luminol Reagent (Santa Cruz Biotechnology) and imaged with a Synopics 4.2 MP camera and G:Box Chemi-XT4 GENESys software (SYNGENE, Frederick, MD). Band density was quantified with Image J software).

### Immunohistochemistry

Immunohistochemistry (IHC) was performed on a DakoCytomation autostainer using the Envision HRP Detection system (Dako, Carpinteria, CA). Each mammary tissue block was sectioned at 4 μm on a graded slide, deparaffinized in xylene, rehydrated in graded ethanols, and rinsed in Tris-phosphate-buffered saline (TBS). Heat induced antigen retrieval was performed in a microwave at 98°C in 0.01 M citrate buffer. After cooling for 20 minutes, sections were rinsed in TBS and subjected to the following primary antibodies: Rat monoclonal anti-BrdU 1:100, (Abcam); Rabbit polyclonal anti-Cleaved Caspase-3 (Asp 175) 1:100 (Cell Signaling); Rabbit polyclonal anti-p53 antibody (CM5) 1:1000 (Leica, Wetzlar, Germany); Rabbit polyclonal anti-PR (C-19) 1:100, (Santa Cruz Technologies) for 45 minutes. Immunoreactivity was visualized by incubation with chromogen diaminobenzidine (DAB) for 5 minutes. Tissue sections were counterstained with hematoxylin, dehydrated through graded ethanols and xylene, and cover-slipped. Images were captured with an Olympus BX41 light microscope using (SPOT™Imaging Solutions, Detroit, MI).

### Statistical analysis

Results were analyzed using a two-way ANOVA with *Sfrp1* loss and HFD treatment as the main effects unless otherwise stated. *Post hoc* tests, where appropriate, were performed by Bonferroni’s *t* test. Bonferroni’s *t* test uses the mean square error from the ANOVA table as a point estimate of the pooled variance (Graphpad Prism, San Diego, CA). Grubb’s test was used on all data to identify statistical outliers (http://www.graphpad.com/quickcalcs). Statistical outliers were identified in some data sets, but the overall results were not altered by omission. A few samples were lost during processes; therefore, there are some unequal sample sizes.

## Abbreviations

Sfrp1: Secreted frizzled related protein 1; DIO: Diet induced obesity; ND: Normal diet; HFD: High fat diet; BrdU: 5-bromo-2-deoxyuridine; Bax: Bcl2 Associated X protein; PUMA: p53 upregulated modulator of apoptosis; Bbc3: Bcl2 bding component 3; Caspase: Cysteine aspartic acid specific protease; RANKL: Receptor of activated NF-κB ligand; Tnfs11: Tumor necrosis factor ligand superfamily member 11; PR: Progesterone receptor.

## Competing interests

The authors do not have any financial or personal relationships with other people or organizations that could inappropriately influence the work described in this manuscript.

## Authors’ contributions

KG drafted the manuscript and performed all of the described experiments with the exception of the immunohistochemistry. AS provided our laboratory with the *Sfrp*^*−/−*^ mice. LB and EH contributed to the mouse work. JW and JS processed the tissues and carried out the immunohistochemistry. SS participated in the study design, edited the manuscript, and gave final approval of the version to be published. All authors read and approved the final manuscript.

## Supplementary Material

Additional file 1: Figure S1 Validation of Sfrp1 mutation and Sfrp1 mRNA loss in the mammary glands of *Sfrp1*^*-/-*^ mice. **(A)** PCR analysis of tail DNA from breeding pairs used to generate mice for the experiments described in the manuscript. Gel electrophoresis revealed that the *SacII*f and *SacII*r primer set yielded a 510-bp wild-type specific fragment by PCR in *Sfrp1*^+/+^ and *Sfrp1*^+/-^ mice and the LacZf and LacZr primer set yielded a 364-bp fragment in *Sfrp1*^+/-^ mice and *Sfrp1*^-/-^ mice as well as all breeders used for the study. **(B)** Total RNA was harvested from the 5th inguinal mammary glands of mice used in the described experiments and employed for real-time PCR analysis of *Sfrp1* gene expression (n = 6/genotype). The results shown represent experiments performed in duplicate and are normalized to the amplification of β*-Actin* mRNA. Bars represent mean ± SEM of the difference in fold change compared with control mice. (***p < 0.05, significantly different from control mice using student’s t-test). Click here for file

## References

[B1] Huang Z Willett WC Colditz GA Hunter DJ Manson JE Rosner B Speizer FE Hankinson SE Waist circumference, waist:hip ratio, and risk of breast cancer in the nurses’ health study Am J Epidemiol 1999 150 1316 1324 10.1093/oxfordjournals.aje.a009963 10604774

[B2] Hanahan D Weinberg RA The hallmarks of cancer Cell 2000 100 57 70 10.1016/S0092-8674(00)81683-9 10647931

[B3] Gauger KJ Hugh JM Troester MA Schneider SS Down-regulation of sfrp1 in a mammary epithelial cell line promotes the development of a cd44high/cd24low population which is invasive and resistant to anoikis Canc Cell Int 2009 9 11 10.1186/1475-2867-9-11 PMC2687411 19422694

[B4] Gauger KJ Shimono A Crisi GM Schneider SS Loss of SFRP1 promotes ductal branching in the murine mammary gland BMC Dev Biol 2012 12 25 10.1186/1471-213X-12-25 22928951 PMC3482146

[B5] Gauger KJ Schneider SS The tumor supressor sectrete frizzled related protein 1 regulates p53-mediated apoptosis Cell Biol Int 2014 38 124 130 10.1002/cbin.10176 24038862

[B6] Gauger KJ Bassa LM Henchey EM Wyman J Bentley B Brown M Shimono A Schneider SS Mice deficient in Sfrp1 exhibit increased adiposity, dysregulated glucose metabolim, and enhanced macrophage infiltration PLoS One 2013 8 e78320 10.1371/journal.pone.0078320 24339864 PMC3855156

[B7] Polakis P Wnt signaling and cancer Gene Dev 2000 14 1837 1851 10921899

[B8] Bafico A Gazit A Pramila T Finch PW Yaniv A Aaronson SA Interaction of frizzled related protein (FRP) with Wnt ligands and the frizzled receptor suggests alternative mechanisms for FRP inhibition of Wnt signaling J Biol Chem 1999 274 16180 16187 10.1074/jbc.274.23.16180 10347172

[B9] Satoh W Gotoh T Tsunematsu Y Aizawa S Shimono A Sfrp1 and Sfrp2 regulate anteroposterior axis elongation and somite segmentation during mouse embryogenesis Development (Cambridge, England) 2006 133 989 999 10.1242/dev.02274 16467359

[B10] Mori H Prestwich TC Reid MA Longo KA Gerin I Cawthorn WP Susulic VS Krishnan V Greenfield A Macdougald OA Secreted frizzled-related protein 5 suppresses adipocyte mitochondrial metabolism through WNT inhibition J Clin Investig 2012 122 2405 2416 10.1172/JCI63604 22728933 PMC3386832

[B11] Ouchi N Higuchi A Ohashi K Oshima Y Gokce N Shibata R Akasaki Y Shimono A Walsh K Sfrp5 is an anti-inflammatory adipokine that modulates metabolic dysfunction in obesity Science (New York, NY) 2011 329 454 457 10.1126/science.1188280 PMC3132938 20558665

[B12] Bodine PV Billiard J Moran RA Ponce-de-Leon H McLarney S Mangine A Scrimo MJ Bhat RA Stauffer B Green J Stein GS Lian JB Komm BS The Wnt antagonist secreted frizzled-related protein-1 controls osteoblast and osteocyte apoptosis J Cell Biochem 2005 96 1212 1230 10.1002/jcb.20599 16149051

[B13] Seol MB Bong JJ Baik M Expression profiles of apoptosis genes in mammary epithelial cells Mol Cells 2005 20 97 104 16258247

[B14] Jiang GX Liu W Cui YF Zhong XY Tai S Wang ZD Shi YG Li CL Zhao SY Reconstitution of secreted frizzled-related protein 1 suppresses tumor growth and lung metastasis in an orthotopic model of hepatocellular carcinoma Dig Dis Sci 2010 55 2838 2843 10.1007/s10620-009-1099-3 20033841

[B15] Cooper SJ von Roemeling CA Kang KH Marlow LA Grebe SK Menefee ME Tun HW Colon-Otero G Perez EA Copland JA Reexpression of tumor suppressor, sFRP1, leads to antitumor synergy of combined HDAC and methyltransferase inhibitors in chemoresistant cancers Mol Canc Therapeut 2012 11 2105 2115 10.1158/1535-7163.MCT-11-0873 PMC3928542 22826467

[B16] Nakano K Vousden KH PUMA, a novel proapoptotic gene, is induced by p53 Mol Cell 2001 7 683 694 10.1016/S1097-2765(01)00214-3 11463392

[B17] Faleiro L Kobayashi R Fearnhead H Lazebnik Y Multiple species of CPP32 and Mch2 are the major active caspases present in apoptotic cells EMBO J 1997 16 2271 2281 10.1093/emboj/16.9.2271 9171342 PMC1169829

[B18] Sharma SD Katiyar SK Leptin deficiency-induced obesity exacerbates ultraviolet B radiation-induced cyclooxygenase-2 expression and cell survival signals in ultraviolet B-irradiated mouse skin Toxicol Appl Pharmacol 2012 244 328 335 20122948 10.1016/j.taap.2010.01.010

[B19] Ford NA Dunlap SM Wheatley KE Hursting SD Obesity, independent of p53 gene dosage, promotes mammary tumor progression and upregulates the p53 regulator MicroRNA-504 PLoS One 2013 8 e68089 10.1371/journal.pone.0068089 23840816 PMC3696069

[B20] Weichhaus M Broom J Wahle K Bermano G A novel role for insulin resistance in the connection between obesity and postmenopausal breast cancer Int J Oncol 2012 41 745 752 22614942 10.3892/ijo.2012.1480

[B21] Imbert A Eelkema R Jordan S Feiner H Cowin P Delta N89 beta-catenin induces precocious development, differentiation, and neoplasia in mammary gland J Cell Biol 2001 153 555 568 10.1083/jcb.153.3.555 11331306 PMC2190562

[B22] Michaelson JS Leder P Beta-catenin is a downstream effector of Wnt-mediated tumorigenesis in the mammary gland Oncogene 2001 20 5093 5099 10.1038/sj.onc.1204586 11526497

[B23] Bradbury JM Edwards PA Niemeyer CC Dale TC Wnt-4 expression induces a pregnancy-like growth pattern in reconstituted mammary glands in virgin mice Dev Biol 1995 170 553 563 10.1006/dbio.1995.1236 7649383

[B24] Faust IM Johnson PR Stern JS Hirsch J Diet-induced adipocyte number increase in adult rats: a new model of obesity Am J Physiol 1978 235 E279 E286 696822 10.1152/ajpendo.1978.235.3.E279

[B25] Suda T Takahashi N Udagawa N Jimi E Gillespie MT Martin TJ Modulation of osteoclast differentiation and function by the new members of the tumor necrosis factor receptor and ligand families Endocr Rev 1999 20 345 357 10.1210/edrv.20.3.0367 10368775

[B26] Fernandez-Valdivia R Mukherjee A Ying Y Li J Paquet M DeMayo FJ Lydon JP The RANKL signaling axis is sufficient to elicit ductal side-branching and alveologenesis in the mammary gland of the virgin mouse Dev Biol 2009 328 127 139 10.1016/j.ydbio.2009.01.019 19298785

[B27] Gonzalez-Suarez E Jacob AP Jones J Miller R Roudier-Meyer MP Erwert R Pinkas J Branstetter D Dougall WC RANK ligand mediates progestin-induced mammary epithelial proliferation and carcinogenesis Nature 2011 468 103 107 20881963 10.1038/nature09495

[B28] Hausler KD Horwood NJ Chuman Y Fisher JL Ellis J Martin TJ Rubin JS Gillespie MT Secreted frizzled-related protein-1 inhibits RANKL-dependent osteoclast formation J Bone Miner Res 2004 19 1873 1881 10.1359/JBMR.040807 15476588

[B29] Fernandez-Valdivia R Lydon JP From the ranks of mammary progesterone mediators, RANKL takes the spotlight Mol Cell Endocrinol 357 91 100 21964466 10.1016/j.mce.2011.09.030 PMC3253322

