# Supplementary figures and images for: The effects of diet induced obesity on breast cancer associated pathways in mice deficient in SFRP1
Source: Mol Cancer. 2014 May 22;13:117. doi: 10.1186/1476-4598-13-117 (PMC4060881; doi:10.1186/1476-4598-13-117)

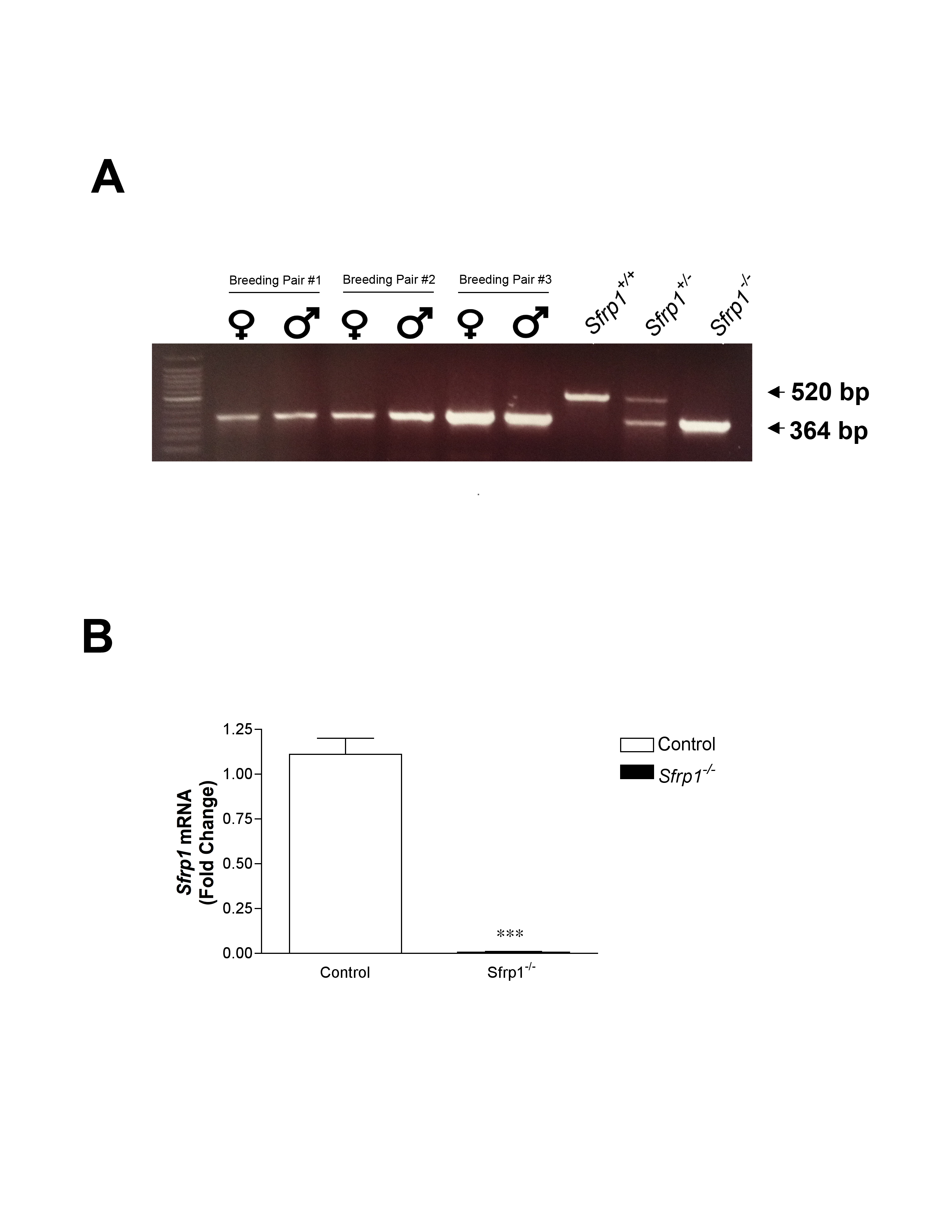

Supplement: Additional file 1: Figure S1 — Validation of Sfrp1 mutation and Sfrp1 mRNA loss in the mammary glands of Sfrp1 -/- mice. (A) PCR analysis of tail DNA from breeding pairs used to generate mice for the experiments described in the manuscript. Gel electrophoresis revealed that the SacIIf and SacIIr primer set yielded a 510-bp wild-type specific fragment by PCR in Sfrp1+/+ and Sfrp1+/- mice and the LacZf and LacZr primer set yielded a 364-bp fragment in Sfrp1+/- mice and Sfrp1-/- mice as well as all breeders used for the study. (B) Total RNA was harvested from the 5th inguinal mammary glands of mice used in the described experiments and employed for real-time PCR analysis of Sfrp1 gene expression (n = 6/genotype). The results shown represent experiments performed in duplicate and are normalized to the amplification of β-Actin mRNA. Bars represent mean ± SEM of the difference in fold change compared with control mice. (***p < 0.05, significantly different from control mice using student’s t-test). [file 1476-4598-13-117-S1.tiff]
